# Nanocarrier-based drug combination therapy for glioblastoma

**DOI:** 10.7150/thno.38147

**Published:** 2020-01-01

**Authors:** Mengnan Zhao, Demian van Straten, Marike L.D. Broekman, Véronique Préat, Raymond M. Schiffelers

**Affiliations:** 1 Université catholique de Louvain, Louvain Drug Research Institute, Advanced Drug Delivery and Biomaterials, Avenue Mounier, 73, B1 73.12, 1200 Brussels, Belgium; 2 Department of Clinical Chemistry and Haematology, University Medical Center Utrecht, Heidelberglaan 100, 3584 CX Utrecht, Netherlands; 3 Department of Neurosurgery, Leiden University Medical Center, PO Box 9600, 2300 RC, Leiden, The Netherlands.

**Keywords:** glioblastoma, nanomedicine, nanoparticles, local delivery, systemic delivery, EPR effect, theranostics

## Abstract

The current achievements in treating glioblastoma (GBM) patients are not sufficient because many challenges exist, such as tumor heterogeneity, the blood brain barrier, glioma stem cells, drug efflux pumps and DNA damage repair mechanisms. Drug combination therapies have shown increasing benefits against those challenges. With the help of nanocarriers, enhancement of the efficacy and safety could be gained using synergistic combinations of different therapeutic agents. In this review, we will discuss the major issues for GBM treatment, the rationales of drug combinations with or without nanocarriers and the principle of enhanced permeability and retention effect involved in nanomedicine-based tumor targeting and promising nanodiagnostics or -therapeutics. We will also summarize the recent progress and discuss the clinical perspectives of nanocarrier-based combination therapies. The goal of this article was to provide better understanding and key considerations to develop new nanomedicine combinations and nanotheranostics options to fight against GBM.

## Introduction

**Figure 1:**
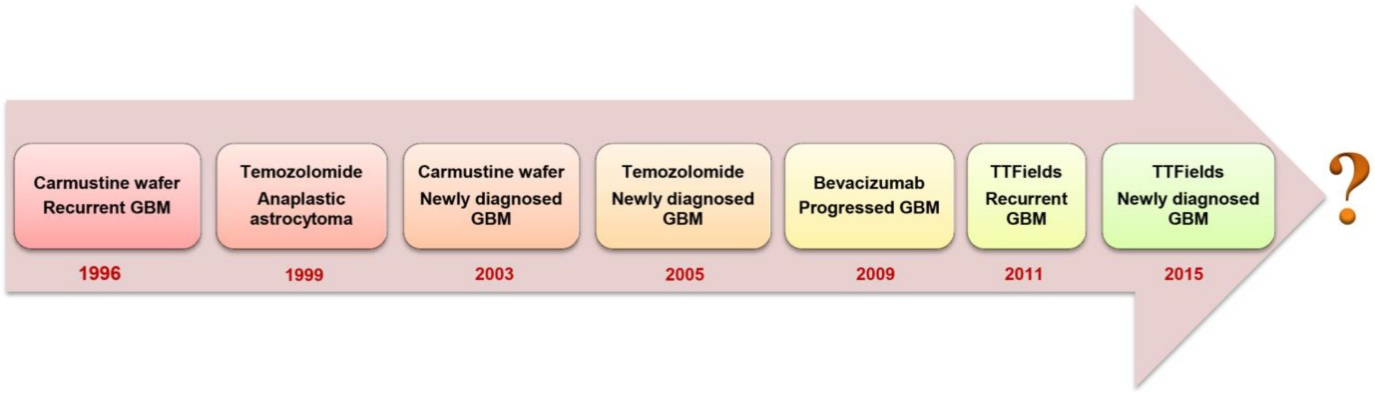
Evolution of FDA-approved GBM treatment approaches.

Glioblastoma multiforme (GBM) is a grade IV malignant glioma associated with very poor patient prognosis 1. Currently, no curative treatment options exist and the 5-year survival of GBM-diagnosed patients remains lower than 6% 2. Upon diagnosis, standard of care involves maximal surgical resection, followed by radiotherapy and concurrent chemotherapy with temozolomide (TMZ) 3. Complete surgical resection is usually unachievable because GBM is only diagnosed when the patient develops symptoms, by which time the highly invasive tumor cells infiltrate to the crucial functional regions of the brain that control senses, actions and speech 4. Although the continuous development of surgical imaging techniques allows increasingly more extensive surgical resections, there is always the need to balance between aggressive removal of tumor tissue whilst maintaining brain function and protecting the life quality of patients 1. A median survival of 12.1 months can be obtained through addition of focal irradiation 3. However, radiation is associated with cognitive impairment, DNA lesions and other severe systemic side effects 5. The anti-angiogenic drug bevacizumab was approved by the U.S. Food and Drug Administration (FDA) to treat recurrent GBM that has progressed after prior therapy. The addition of bevacizumab to concomitant chemo-radiotherapy for newly diagnosed GBM showed prolonged progression-free survival (PFS) but failed to show an overall survival (OS) benefit in phase III trials 6, 7. Optune^®^, a device that creates low-intensity and alternating tumor-treating fields, also obtained approval from the FDA as treatment together with TMZ for newly diagnosed GBM in 2015 8. Despite these multidisciplinary therapies, most patients still develop tumor recurrence within 1 to 2 years of diagnosis. Patients may then undergo repeated resection, different chemotherapies, bevacizumab therapy or additional radiotherapy. Unfortunately, there is limited evidence showing that those treatments can increase the survival time. Thus, regarding the unmet medical needs for GBM patients, the fight against GBM is far from over (Figure 1).

The general concerns associated with chemotherapy include the blood brain barrier, complicated tumor heterogeneity, glioma stem cells, DNA damage repair mechanisms and drug efflux pumps (Table 1 and Graphical abstract) 9, 10.

**Figure 2:**
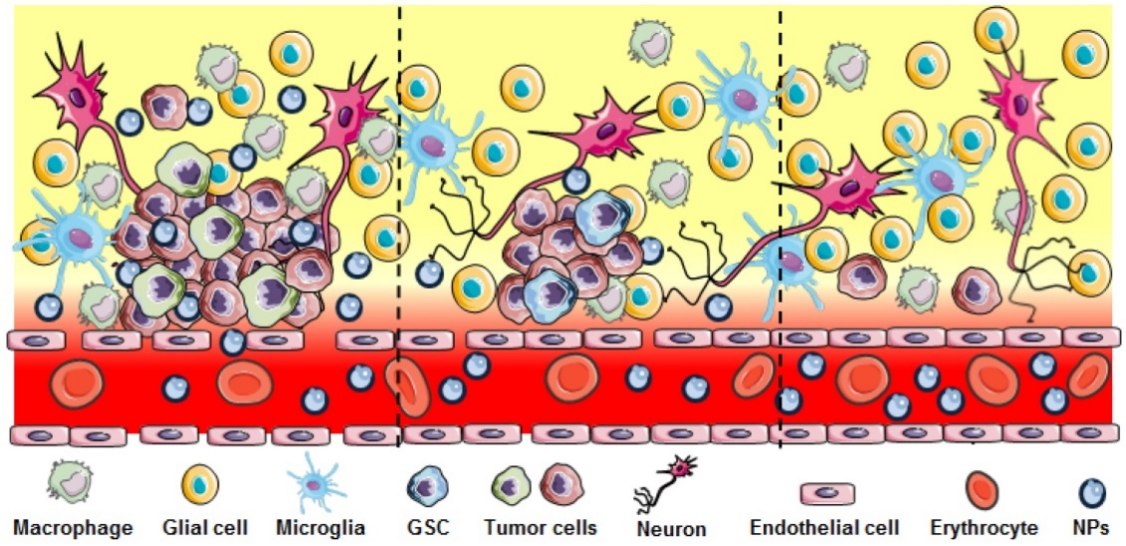
Heterogeneous disruption in GBM. Significant BBB breakdown seen in the bulk tumor region (left panel) allows nanoparticle extravasation. Regions with infiltrating GBM and GSC cells show less or no breakdown of the BBB (middle and right) preventing NPs or other therapeutics to reach these cells.

The blood brain barrier (BBB) is a particularly formidable challenge in developing therapeutics for brain tumors. Brain microvascular endothelial cells, pericytes, astrocytes, tight junctions, neurons and the basement membrane together form a physical barrier to protect the brain and maintain a well-defined intracranial environment 11. Although several specialized transport systems mediate the entry of essential substances such as nucleosides, glucose, amino acids, hormones and receptor-mediated endocytosis *via* specific proteins (e.g., transferrin and lactoferrin), the tight junctions prevent the passive penetration of hydrophilic molecules from the blood circulation to the brain. GBM therapeutics need to be able to cross this barrier and penetrate the brain to reach the tumor. However, only small lipophilic chemotherapeutic agents with a molecular weight less 400 Da and 8 hydrogen bonds can passively pass through the BBB 12. TMZ is an orally administered alkylating agent that can be transported across the BBB and has remarkable distribution at the tumor site. However, TMZ-induced cytotoxic effects can be neutralized by various DNA repair mechanisms, re-enforcing the structural integrity of the methylated DNA bases before causing extensive tumor cell death. High-grade gliomas are characterized by disrupted and heterogeneous blood brain tumor barrier (BBTB) (Figure 2), while the tricky task in GBM treatment is reaching the residual tumor cells infiltrating to brain parenchyma where the BBTB is intact or less compromised, leading to an insufficient therapeutic effect through passive drug diffusion 13.

Another difficulty is found in the heterogeneity of GBM. Genomic research has shown that GBM contains many different cell types depending on their origin or subsequent genetic and epigenetic conversions 14. Single-cell sequencing of five primary GBM showed inherently variable gene expression in diverse transcriptional programs associated with oncogenic signaling, hypoxia, proliferation and the complement/immune response 15.

This genetic drift can result in self-renewing, tumorigenic glioblastoma stem cells (GSCs) that contribute to tumor initiation and therapeutic resistance 16. Stem cell-like properties allow GSCs to differentiate into highly proliferating progenitor-like tumor cells or other differentiated tumor cells, which can be more resistant to radio- and chemotherapy than non GSC tumor cells both *in vitro* and *in vivo*. Consequently, populations of glioma stem cells remain alive after initial treatment and reinitiate tumor recurrence 10.

Multidrug resistance (MDR) presents another major barrier for chemotherapeutic drugs to get access to brain tumor cells effectively. Among the different mechanisms of MDR, ATP-binding cassette (ABC) transporter-mediated exocytosis was mostly noticed. The P-glycoprotein (P-gp) which is encoded by the human *MDR1* gene has been mostly considered as the cause of anti-cancer drug resistance. *MDR1* P-gp is present in the brain capillaries of the BBB, as well as in many other tissues. Many drugs exhibit significantly improved brain penetration when drug efflux transporters are inhibited 17.

Because of these challenges, combining drugs with different working mechanisms has gained great attention in recent years. The right combination of compounds could enhance efficacy by targeting these issues in a synergistic or additive manner. However, the efficiency of many chemotherapeutic agents is also limited by their dose-related toxicities. As the BBB shields the brain from most systemically administrated compounds, high doses are given to achieve intracranial therapeutic drug levels. Increasing the dose of a specific anticancer drug will inevitably lead to significant toxicity. Many GBM chemotherapeutic drugs have demonstrated off-target toxicity at the doses needed to reach an intracranial effect. For example, TMZ is associated with lymphopenia, thrombocytopenia and neutropenia 18 and bevacizumab is frequently associated with hypertension, leukopenia, non-central nervous system hemorrhage and thromboembolic events 19. Thus, combining drugs with non-overlapping toxicities and reducing the dose of each single drug may be a better choice.

With growing investigation of the tumor microenvironment and by unravelling biological and molecular pathways, increasingly more potential drug combinations are emerging. However, just combining cytotoxic compounds does not address the problems associated with poor drug distribution at the desired tumor site. Different approaches have been raised to defeat unfavorable drug distribution in the brain 20. Among these, nanotechnology-based drug delivery is a promising strategy to enhance chemotherapy efficiency. Various nanocarriers have been investigated for drug delivery in central nervous system (CNS) tumors, such as polymeric nanoparticles, liposomes, and lipid nanocapsules. The correct nanocarrier could enhance the solubility of hydrophobic drugs, prolong compound circulation times and provide sustained drug release, improving therapeutic efficacy and safety 21, 22. They can be administered locally or systemically, with the potential benefit of the enhanced permeation and retention (EPR) effect.

In this review, we will discuss and address the advantages of drug combinations with or without nanocarriers, nanomedicine-based tumor targeting strategies, current preclinical drug combinations, and promising nanotherapeutics and nanodiagnostics. The aim of this review was to highlight the importance and potential of drug combinations and give a comprehensive understanding of different combination strategies for GBM therapy.

## Drug combination strategies

Poor drug delivery, tumor heterogeneity and drug resistance pathways have prevented single compound therapies to show significant benefits for GBM patients 2. Combining drugs could overcome some of the problems associated with GBM treatment. Ideally, drug combinations take advantage of each individual compound's strengths and weaknesses to improve efficacy, decrease toxicity and overcome drug resistance. It starts with the method of administration (systemic versus local), which can heavily influence these parameters and determines how each compound is delivered.

### Systemic delivery

Drug delivery to GBM is notoriously difficult due to the inability of most drugs to cross the BBB and penetrate the tumor tissue. Only few systemically administrated drugs reach the tumor site in a therapeutic dose. Various approaches, such as chemical modification of chemotherapeutic drugs, BBB altering strategies and efflux transporter inhibitors, are being investigated to enhance the systemic delivery of potential anti-GBM drugs. For instance, drugs can be modified to a more lipophilic form by adding lipid groups to the polar ends of therapeutic molecules. A log P (octanol-water) value ranging from 1.5 to 2.5 of lipophilic analogs has better brain permeability 23. However, this may also increase nonspecific uptake of the drug molecule by other tissues through the blood circulation.

Alternatively, hyperosmotic agents, bioactive molecules, surfactants and ultrasound or electromagnetic waves have been used to alter the permeability of the BBB. Yet, such approaches are often associated with risks such as possible tumor diffusion to the periphery, and exposure of the brain to neurotoxins.

In addition, inhibition of ABC efflux gene families can increase drug penetration into the brain without compromising the integrity of the tight junctions and endothelial layers. However, this approach will also reduce the efflux of potential neurotoxic compounds. In fact, many of these efflux pumps transporters could not be fully inhibited due to various reasons, including multifactorial multidrug resistance and genomically unstable tumor cells 24. Thus, further investigations of various systemic delivery approaches through enhancing BBB permeability are still required to achieve a significant therapeutic effect for GBM treatment.

### Local delivery

Local intracranial delivery not only overcomes BBB-associated drug delivery issues but also prevents systemic compound clearance and/or degradation and reduces systemic side effects. As such, much lower dosages are needed. Local drug delivery to the brain and further distribution within the brain can be mediated by simple diffusion using a reservoir-catheter system or positive pressure bulk flow *via* convection enhanced delivery. The Ommaya reservoir is a reservoir capsule connected to a catheter located in the lateral ventricle. The capsule is embedded under the scalp and is easily accessible for cerebrospinal fluid (CSF) aspiration or drug delivery directly into the ventricular CSF and relies on the flow of CSF to distribute chemotherapeutics, radioactive compounds, antibodies, viruses or cells throughout the brain 25.

Alternatively, drugs are administered to the brain continuously with a positive pressure bulk flow *via* convection-enhanced delivery (CED). The positive pressure drives convective local transport of therapeutic concentrations of anti-tumor drugs into the interstitial tumor space. This technique achieved higher drug concentrations in the targeted tumor tissue compared with diffusion-limited delivery 26. Unfortunately, common side effects, such as edema, infection and backflow along the catheter have resulted in the limited application of CED to treat GBM 26.

Locally implanted (biodegradable) drug delivery depots have increasingly gained interest. Currently, the only FDA-approved biodegradable implant is the Gliadel^®^ wafer for newly diagnosed malignant and recurrent GBM. However, the success of Gliadel^®^ wafers is restricted by the limited penetration of the active compound, carmustine, into the brain tumor tissue. Moreover, use of the wafers has been associated with several adverse events, including intracranial infections, wafer migration, cerebral edema, CSF leakage and seizures 27.

Nevertheless, the concept of local delivery by implanting drug-releasing depots in the tumor resection cavity remains intriguing for the treatment of GBM. Films, foams and gels have been investigated for their use in local drug delivery. Particularly hydrogels have gained much attention in recent years. In general, hydrogels are injectable, biocompatible, biodegradable and mechanically comparable to soft tissue, making them attractive for intracranial implantation. They provide a versatile drug delivery system because they can be loaded with small-molecule drugs, biomacromolecules (such as DNA or protein) or cells. The gels can be engineered to tune the release of their contents in a timeframe ranging from hours up to several months 28. A hydrogel composed of gemcitabine lipid nanocapsules obtained sustained drug release for at least 1 month and significantly delayed tumor recurrence and prolonged survival in a GBM resection mouse model 29. This hydrogel was also able to co-deliver gemcitabine and paclitaxel (PTX). The drug combination was shown to be synergistic in different GBM cell lines 30. Similarly, local treatment of GBM with a photopolymerizable hydrogel coloaded with PTX and TMZ suppressed tumor growth more efficiently than the single drugs in an orthotopic U87MG tumor resection model 31. Taken together, local delivery appears to be an effective approach to improve chemotherapy-based treatment for GBM by increasing the local dose of chemotherapeutics and simultaneously reducing systemic side effects. Polymeric implants and hydrogels show great promise but need additional development and optimization before they can be translated to clinical practice 32.

### Recent clinical trials of drug combinations for GBM treatment

Different drug combination strategies have been explored in clinical trials to tackle known drawbacks of GBM treatment. A non-exhaustive summary of combination therapy trials is presented in table 2 (Source: ClinicalTrials.gov). Several chemotherapeutics have been combined with anti-angiogenic drugs. A phase II clinical trial using bevacizumab and the topoisomerase inhibitor irinotecan showed a 6-month PFS rate of 50.3% compared to 42.6% with bevacizumab alone in recurrent GBM. The median overall survival times were 9.2 months and 8.7 month for combination therapy and bevacizumab alone respectively 33.

Another phase II clinical trial combined TMZ with an O^6^-methylguanine-DNA methyltransferase (MGMT inhibitor) O^6^-benzylguanine (O^6^-BG) to rebuild drug sensitivity in TMZ-resistant anaplastic glioma. Indeed, O^6^-BG was able to restore TMZ sensitivity in TMZ-resistant anaplastic glioma, but not in TMZ-resistant GBM 34.

More often than not, promising preclinical anti-tumor strategies disappoint in clinical trials due to various reasons. For example, a phase I/II trial to determine the efficacy of vorinostat + erlotinib versus vorinostat + erlotinib + TMZ in patients with recurrent GBM multiforme was terminated because of unanticipated toxicities (NCT01110876). A trial that combined the histone deacetylase inhibitor vorinostat and proteasome inhibitor bortezomib to treat recurrent GBM, reported that no patient achieved 6-month PFS and only one patient showed a partial response according to the Macdonald Criteria, probably due to brain delivery issues 35.

The underwhelming results of drug combinations in clinical trials compared to the encouraging *in vitro* results can be explained by several factors (table 1). In some cases, the ratio between the compounds is important for their combined efficacy. *In vivo*, the differences in drug distribution, metabolism and excretion for each single drug need to be considered and fine-tuned to realize the desired local concentrations. Dose-limiting toxicity and, although rarely reported, insufficient intracranial drug accumulation, seem to explain most of the disappointing trials. This appears to be surmountable as systemic toxicity and increased brain penetration of drugs can be addressed by alternative delivery strategies.

## Nanocarrier-based combination therapy for GBM

Preclinical and clinical research has shown that various factors may compromise the efficacy of (combinations of) therapeutics, which has led to disappointing clinical outcomes (table 1). For GBM, the BBB seems to be the main dissonant. In recent years, nanomaterials have gained attention as they have the potential to overcome many of these hurdles, mask unfavorable characteristics of the active compounds and/or improve their efficacy. It is estimated that due to the BBB, 100% of large molecules and 98% of small molecules fail to sufficiently reach the brain to achieve therapeutic levels 36. Nanoparticles (NPs) encapsulating these molecules can be tailored to enable specific transport of their payload to the brain or facilitate penetration through the BBB, thereby enabling encapsulated drugs for previously unreachable tumors such as GBM 37.

Depending on the materials, nanoformulations are able to load hydrophilic and hydrophobic drugs, ensure sustained drug release and enhance the half-life of the drug in the circulation. For example, the half-life of TMZ was enhanced to 13.4 h compared to 1.8 h of the free drug by encapsulation in a chitosan-based nanoparticle 38. Improving compound solubility, stability and reducing systemic toxicity are major goals in designing such formulations. Several FDA approved nanoformulations (e.g. Abraxane^®^, Doxil^®^, DaunoXome^®^) mainly reduce toxicity of the parent compound and thereby improve its therapeutic index. Small interfering RNAs (siRNA) show great therapeutic promise but have disappointing clinical relevance due to stability and delivery issues. The only currently FDA approved siRNA therapeutic is Patisiran^®^, which is based on a lipid NP formulation that improves siRNA stability.

Two decades have passed since the first NP-based cancer treatment was approved by the FDA, with an increasing number of clinical trials now ongoing, including many for GBM. A variety of systems based on polymers (micelles, dendrimers), inorganic materials (iron, silica, gold) and/or lipids (liposomes, solid lipid nanocarriers) have been investigated for drug delivery to the brain. The therapeutic drugs can be loaded in these particles by encapsulation, covalent linking or surface adsorption 37. Depending on the design of the drug delivery system, the drugs are either passively or actively targeted to the tumor to exert their effect.

### Passive targeting

Passive tumor targeting is based on the observation that certain sized particles tend to accumulate in tumor tissue much more than they do in healthy tissues. This is known as the enhanced permeability and retention effect (EPR) effect, which was first described by Matsumura and Maeda in 1986 39. EPR is based on aberrant pathophysiological characteristics of tumors. The presence of abnormal, fenestrated vasculature and a lack of proper lymphatic drainage results in the extravasation of NPs and reduced lymphatic clearance 40, 41 .

**Figure 3:**
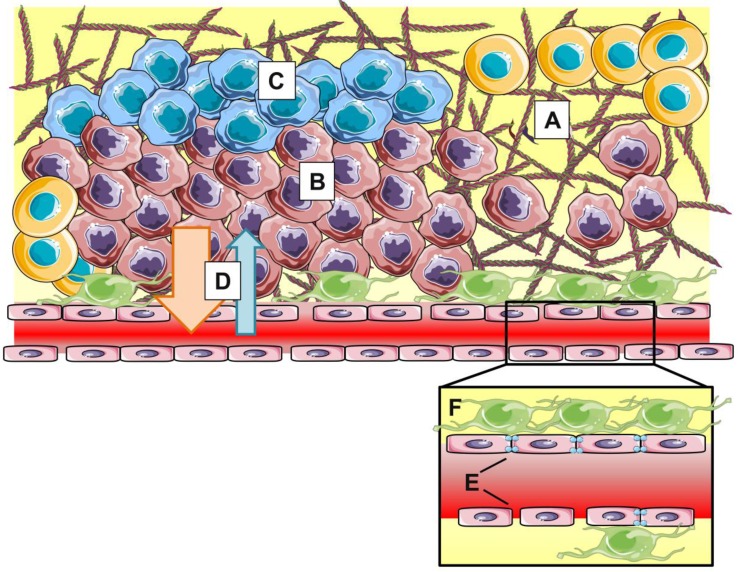
The EPR effect is influenced by stromal parameters such as dense extracellular matrix (A), hypercellularity (B), hypoxia (C) and high interstitial fluid pressure (D). At blood vessel level (insert), heterogeneity in vascular permeability, tight junction expression (E) and pericyte coverage (F) result in varying clinical manifestations of EPR.

However, within the last couple of years, more and more researchers have realized that the EPR effect is highly heterogeneous both intra- and intertumorally, varies during tumor development and does not always hold up in clinical settings (Figure 3). Moreover, the magnitude of the EPR effect as seen in rodent models fails to translate to the clinic. Human tumors are drastically different from preclinical tumor models in many critical aspects such as: (*i*) heterogeneity or lack of fenestrations in the tumor endothelium, (*ii*) presence of acidic and hypoxic areas (*iii*) lower or heterogeneous pericyte and basement membrane coverage, (*iv*) and high interstitial fluid pressure (IFP) induced by dense extracellular matrix, explaining the difficulty in translating the EPR effect from bench to bedside 42.

Interestingly, GBM is characterized by robust endothelial proliferation resulting in tortuous, disorganized and highly permeable vasculature 43, 44. Such excessive neovascularization also affects BBB integrity which can be visualized by magnetic resonance imaging (MRI). Contrast agents such as gadolinium do not cross a healthy BBB, but in pathologies such as GBM, hypervascularization causes physical disruptions of the BBB that allow leakage of gadolinium into the tumor tissue, making the tumor visible on T1-weighted MRI. Gadolinium-enhanced areas form the golden standard in GBM diagnosis and provide guidance for surgical resection. Thus, theoretically, intravenously administered nanocarriers could exploit this phenomenon in GBM. Yet, it is evidently not this simple in the clinic.

First of all, when comparing different imaging modalities, it becomes clear that T1-weighted MRI alone does not visualize the entire tumor. Beyond the contrast-enhancing region, essentially all of GBM show non-enhancing edema on T2-weighted or fluid attenuation inversion recovery imaging 45. These areas have impaired fluid regulation yet do not accumulate contrast agent, suggesting that the BBB is intact. Additionally, the impaired fluid regulation leads to high interstitial fluid pressure which compromises transvascular transport of molecules. It is suggested that the invasive tumor cells and tumor associated stromal cells in this peritumoral brain zone drive 90% of all recurrences 46, 47. These cells are not or barely reached by passively targeted drugs or NPs due to their location behind an intact BBB. This should be considered when designing new therapies and drug delivery systems (DDSs), as relying solely on the EPR effect may be insufficient to target these cells 24, 44, 46.

Other key pathological features of GBM are hypoxic areas surrounded by hypercellular rings of actively migrating tumor cells 48. These hypoxic zones, arising from inadequate vascularization, cannot be adequately reached by NPs or therapeutics in general. Moreover, hypoxia gives rise to a group of so-called pseudopalisading tumor cells that are highly migratory, pro-angiogenic, therapy resistant and show decreased proliferation 48-50.

Due to the differences in EPR effect between and within tumors, approaches to still take advantage of it may vary. In certain situations, one might benefit from tumor vasculature normalization 51-53, while in others, increasing vascular leakiness or opening up the BBB will improve treatment 54, 55. Non-steroidal anti-inflammatory drugs (NSAID) such as COX-inhibitors are able to reduce BBB disruption in neuro-inflammation 56 and prevent protein extravasation in glioma 57. COX-2 inhibition normalized the tumor microenvironment including its vasculature and improved the penetration and accumulation of micelles (22 nm) rather than bigger (100 nm) NPs in a solid tumor mouse model 58. Similarly, reducing the leakiness of tumor vasculature by blocking the VEGF receptor, improved the delivery of nanoparticles in a size dependent manner in a mammary mouse model 59. In contrast, the A2A adenosine receptor agonist lexiscan is approved by FDA for myocardial perfusion imaging. Lexiscan has the ability to transiently open the BBB and enhance the permeability of NPs to the brain 60. Integrating lexiscan in NPs improved its BBB traversing properties and increased the efficacy of encapsulated therapeutics in an orthotopic glioma mouse model 61. Focused ultrasound (FUS) in combination with circulating microbubbles has been demonstrated to be an effective approach to locally increase BBB permeability. The delivery of PTX-liposomes to mice brain tissue could be effectively improved by pulsed FUS sonication resulting in a two-fold higher local drug concentration and improved survival in an intracranial mouse GBM model 62.

The extent of EPR in a tumor could be tested prior to treatment using imaging modalities. In a preclinical study, the relative blood volume was found to correlate with the tumor accumulation of polymeric drug carriers. With the help of contrast-enhanced ultrasound imaging, this vascular parameter proved useful in predicting EPR-mediated tumor accumulation of NPs and could possibly be used to preselect patients eligible for nanotherapeutic treatment 63. The size of NPs would affect their tumor distribution as well. In one study, ^64^Cu-labeled long-circulating particles of different size were systemically administered and tracked *via* MRI and positron-emission tomography (PET) in an intracranial rat GBM model. It was demonstrated that 7 hours after systemic injection, the uptake of 20 nm NPs is significantly greater than those of 110 nm. It also showed that PET/MRI co-registration of brain images may contribute to the monitoring of disease progression and determining what drug delivery approach is feasible 64.

The high variability of the EPR effect is considered as one of the major challenges for translating nanomedicine into the clinic. This issue can be addressed by indirect EPR imaging, utilizing companion diagnostics or developing systems with both diagnostic and therapeutic properties (theranostics). Indirect EPR imaging can non-invasively visualize and quantify the key EPR-determining parameters of the tumor vasculature, while theranostics or companion diagnostics can give insight in NPs distribution and tumor responses.

### Tumor immune microenvironment and nanocarrier-based drug delivery

Many cancers are preceded by infections or (chronic) inflammation and it is increasingly clear that the immune system plays a central role not only in cancer development but also in tumor progression 65. In GBM, the tumor immune microenvironment heavily influences progression, invasion, metabolic reprogramming and therapy resistance, mostly orchestrated by the present immune cells 66. Peripheral monocytes are attracted by tumor secreted factors, infiltrate the brain, differentiate to macrophages and together with the residential microglia develop a class of cells called tumor associated microglia/macrophages (TAM) 67. They can represent up to 50% of the GBM mass and high TAM density has been correlated with glioma grade 68 and poor prognosis 69. GBM cells produce and secrete chemoattractants and signaling molecules to create an environment that drives TAM towards a predominantly immunosuppressive and tumor supportive (TAM2), rather than an immune-stimulatory and anti-tumor phenotype (TAM1) 70. Immunosuppressive TAM promote tissue remodeling and angiogenesis, thereby driving GBM progression 67. Additionally, TAM further drive angiogenesis *via* the secretion of pro-angiogenic factors such as vascular endothelial growth factor (VEGF) and CXC-chemokine ligand 2, paving the way for hypervascularity as seen in GBM 71. Modulation of the TAM polarization has been emerging as a new therapeutic target for GBM. Polarization from TAM2 towards TAM1 not only activates the cytotoxic T cells but also induces the secretion of antitumor cytokines. Zhao *et al.* developed an albumin-based biomimetic NPs with transferrin receptor-binding peptide T12 and mannose as targeting ligands for codelivery of the disulfiram/copper complex and the macrophage modulator regorafenib. The T12 peptide can enhance BBB permeability and glioma cell uptake. The mannose ligand can bind to mannose receptors on TAM2. This system efficiently inhibited the glioma cell proliferation, successfully induced the protumor TAM2 towards antitumor TAM1 and triggered macrophage-directed anti-glioma immunotherapy *via* TAM, regulatory T cells, CD8+ T cells and cytokines 72.

The inflammatory response observed in GBM results in increased vascular permeability as well. In turn, this will not only allow for extravasation of immune cells, but also of therapeutics including NPs. Furthermore, extracellular matrix (ECM) remodeling can influence NPs transport through the interstitial space 73. Second, in addition to modulation of the physical tumor environment (e.g. ECM and vasculature), TAM can also directly contribute to drug retention. Macrophages protect the body by specializing in phagocytosing pathogens, cellular debris and foreign materials. It can be expected that the bulk of NPs that are taken up by the tumor ends up in these cells 74. Indeed, most NPs end up in TAM, even when they only represent 1% of tumor mass 75. Elegant studies by Miller *et al*. show that NPs predominantly accumulate in tumor associated immune cells rather than tumor cells. They used fluorescently labeled polymeric NP (100 nm) together with magnetic NP (20 nm) in several tumor mouse models to predict NP tumor accumulation and treatment outcome. It appeared that NP distribution was mainly determined by vascularization and permeability at early time points, but at later time points by cellular uptake. Both NPs were mostly taken up by host phagocytes (> 90%). High local TAM counts correlated with increased NP accumulation and as such proved an important component of the EPR effect 76. Interestingly, the same group showed that NP accumulating TAM can function as a drug depot, which slowly releasing encapsulated therapeutics to surrounding (tumor) cells. Depleting TAM numbers reduced NP accumulation and treatment efficacy, illustrating a role for TAM in tumor drug retention 77. Furthermore, it was observed that phagocytes, after ingestion of NPs, can move across the vascular wall and carry the NP to the extravascular space. *In vitro* studies show monocyte mediated transfer of NP over an endothelial monolayer 78. *In vivo*, TAM with ingested NPs has been observed to cross the BBB and migrate to (distant) tumors. As such, NPs were shuttled between contralateral CNS tumors *via* migrating TAMs 79. Thus, TAMs are able to modulate the tumor microenvironment thereby enhancing vascular and interstitial permeability, while their phagocytic nature drives NP uptake and prolongs retention in the tumor. Since TAMs are key regulators of several EPR driving mechanisms, it seems natural to investigate ways to take advantage and try to improve drug delivery *via* these cells.

Another point that might influence the immune microenvironment-based therapy for GBM is the immunosuppressive effects of chemotherapy. Indeed, systemic chemotherapy might suppress the bone marrow and consequently impact the amount and activation state of immune cells 80. Nevertheless, some promising results have been seen when giving local chemotherapy. It is exciting to note that local chemotherapy was able to potentiate anti- programmed death 1 (PD-1)-mediated antitumor immune response through increased percentage of dendritic cells, greater antigen presentation and further clonal activation of tumor-specific T cell responses 81. Additionally, it was shown that cancer cells may undergo bona fide immunogenic cell death (ICD) after exposure to some chemotherapeutics that are currently used in clinic. This process generates specific changes in cell surface structures and releases soluble mediators, *e.g.* ATP, calreticulin, high-mobility group box 1(HMGB1) and chemokine ligand 10 (CL10), allowing dendritic cells to recognize the dying cell and initiate an anti-tumor immune response to clear tumor cells 82. Further investigations may focus on nanoimmunotherapy in combination with the optimized dosing of chemotherapeutic drugs.

### Active targeting

When passive targeting is insufficient, active targeting of NPs can facilitate their transport over the BBB. With polymeric NPs this is often achieved by modifying their surface with surfactants or BBB- or glioma-specific ligands. For example, doxorubicin (DOX) loaded poly butyl-cyanoacrylate NPs were more effective when coated with the surfactant P80 and increased the survival time of GBM tumor bearing rats compared to the non-coated group 83. Additionally, conjugation of the BBB ligand transferrin to PTX loaded poly(lactic-co-glycolic acid (PLGA)-NPs showed significant enhancement of cellular uptake and cytotoxicity on C6 rat glioma cell line, in comparison with the non-conjugated PLGA NPs, by taking advantage of receptor mediated endocytosis (RME) 84. Similarly, targeting polymeric micelles grafted with cyclic Arg-Gly-Asp (cRGD), a ligand with selective affinity for the αvβ3 and αvβ5 integrins that are overexpressed on tumor vasculature and tumor cells, vastly enhanced micelle uptake in tumor in an orthotopic mouse GBM model. Compared to specific targeted micelles, RGD-micelles loaded with oxiplatin significantly reduced tumor growth 85.

Lipid NPs, liposomes in particular, are also widely investigated in the drug delivery field. Liposomes can encapsulate hydrophilic compounds in the aqueous compartment and hydrophobic compounds in the bilayer making it a versatile delivery vehicle. Covering the surface of these particles (or NPs in general) with polyethylene glycol (PEG) neutralizes surface charge but thereby ensures prolonged circulation and increases the EPR effect. Radiotherapy supplemented with PEGylated liposomal DOX (Caelyx^®^) resulted in a significantly higher intratumoral concentration of DOX than normal brain tissue in GBM patients 86. Positively charged liposomes have been shown to penetrate BBB by electrostatic interaction with polyanions on the BBB, which leads to adsorptive-mediated endocytosis 87. Alternatively, the liposomal surface can be decorated with targeting ligands to facilitate BBB transport *via* RME and promote tumor specific uptake. Transferrin conjugated liposomes increased brain delivery of 5-fluorouracil by 13 times compared to non-conjugated liposomes 88. In another study, interleukin-13-grafted liposomes significantly enhanced the cytotoxicity and tumor accumulation of DOX in comparison with the free drug on a subcutaneous mouse glioma model 89.

Lipid nanocarriers (LNCs) are also able to load both hydrophilic and hydrophobic molecules in an aqueous core by formation of reversed micelles 90. Similar to liposomes, LNCs may also be coated with PEG or grafted with ligands to ensure efficient brain drug delivery 91. One LNC component, the non-ionic surfactant HS15, was indicated as a key element in producing a P-gp suppressing effect which could aid retaining the delivered therapeutic in the brain 92.

Sometimes NPs are made by organic-inorganic hybrid materials as the magnetic and optical features of metallic NPs can be used to actively target to tumor site and monitor delivery non-invasively. In an U87MG orthotopic tumor model, magnetic targeting treatment with superparamagnetic iron oxide (SPIO) and PTX coloaded PLGA NPs significantly enhanced the median survival time compared with the passive targeting treatment group 93. The tunable small size of gold NPs makes them good candidates as carriers for delivering cargoes across the BBB and targeting brain tumors. Transactivator of transcription (TAT) peptide-targeted multifunctional gold NPs were able to efficiently cross the BBB in an intracranial GBM mouse model and deliver anticancer drug DOX and gadolinium contrast agents to brain tumor tissues 94. Chlorotoxin (CTX) has been shown to be a specific target and efficacious in blocking the glioma Cl channel activity. In one publication, a ^131^I-labeded CTX-functionalized polyethylenimine-entrapped gold nanoparticles as a multifunctional glioma-targeting nanoprobe was generated. After incubation with the NPs, C6 cells displayed much stronger fluorescence intensities than those treated with negative controls under the same conditions by confocal imaging. This CTX-loaded NP could also act as a nanoprobe for the targeted single photon emission computed tomography (SPECT)/ computed tomography (CT) imaging of glioma cells and in a subcutaneous tumor model 95. In another study, the PLGA-gold hybrid NPs were loaded with docetaxel and targeted using angiopep-2. Targeting the NPs improved the efficacy of docetaxel due to improved delivery, while the gold allowed X-ray imaging of the accumulated NPs in a xenograft mouse GBM model. Moreover, thermal therapy could be applied by exposing the gold NPs to an 808 nm laser, which further increased therapeutic efficacy through local heating 96.

Overall, NPs show great potential in preclinical studies. They often improve accumulation of therapeutic compounds *via* passive targeting, while active targeting might be employed to increase this accumulation in hard to reach tumors such as GBM. Imaging (whether direct or indirect) can give an indication of the potential of certain tumors to be treated with NP formulations, thereby preselecting patients and possibly improving treatment success.

### Nanocarrier-loaded with diagnostic or theranostic agents

Nanocarrier formulations additionally offer the opportunity to incorporate imaging features facilitating diagnostic as well as monitoring features. Accurate diagnosis is essential for adequate cancer treatment. For GBM, imaging is pivotal as the alternative diagnostics (e.g. biopsies or surgery) are highly invasive. As such, imaging is crucial for (preliminary) tumor characterization and localization, planning of surgical strategies and monitoring of treatment response.

Generally, maximal gross resection is correlated with increased survival 97, 98. Maximum safe resection is executed based on preoperative imaging combined with intraoperative image-guided surgery. Contrast enhanced MRI is the mainstay imaging technique of GBM, sometimes supported with PET or CT. They are invaluable yet suffer from drawbacks such as limited sensitivity (tumor vs healthy tissue), discriminative power (pseudoprogression vs progression), low anatomical information, hazardous radiation and contrast agent delivery issues (as discussed earlier in this review) 99, 100. As such, there is a constant search to improve technologies and methodologies to push neuroimaging forward. In recent years more advanced techniques such as dynamic susceptibility contrast (DSC)-MRI 101, dynamic contrast enhanced (DEC)-MRI 102 and amino acid PET 100 allow for more accurate imaging of GBM as well as monitoring treatment response.

Where conventional contrast enhancers and tracers can only enter the brain *via* compromised and leaky vasculature, amino acid linked PET tracers can transverse the intact BBB 103. Subsequent tumor accumulation of these tracers occurs due to the increased metabolic demands, hyper vascularity and overexpression of specific transporters in neoplastic tissue 104. Similar to amino acids, NPs can help to cross the BBB and improve tumor localization and simultaneously offer a platform for additional imaging probes or other molecules such as targeting ligands. Moreover, nanoformulations can increase tracer circulation times which proved beneficial for their clinical relevance 105. It is therefore of no surprise there is growing interest in designing diagnostic NPs and further improving brain tumor imaging 106.

Coupling the integrin targeting ligand RGD to PET tracers increased their tumor specificity in mouse models 107 and allowed more accurate tumor to background distinction and treatment response monitoring 108, 109. Tumor localization of RGD coupled-tracers was also seen in patients but seemed to be hampered by intact BBB and partial tumor volume issues 110. Nevertheless, RGD-tracers were able to detect GBM lesions and predict treatment response to chemo-radiotherapy in patients 111. Similarly, a gastrin-releasing peptide receptor targeted gadolinium tracer was conjugated with the near infrared fluorophore IRDye800CW forming a dual modality PET/near infrared (NIR) tracer which allowed intra-operational NIR image-guided resection in human GBM patients 112.

Preclinically, novel materials and approaches are being investigated such as quantum dots (QDs), metallic NPs, protein conjugates and polymeric particles 113. QDs are semi-conducting nanocrystals (2-10 nm) with superior optical properties such as strong resistance to photobleaching, broad excitation spectra with narrow emission spectra, and long fluorescent lifetime 114. Therefore they hold great promise in the field of fluorescent imaging of tumors. QDs functionalized with a tumor penetrating peptide showed increased localization to intracranial GBM tumor tissue compared to healthy brain tissue in mice, mostly due to EPR effect. The QDs could be visualized in the tumor tissue *via* fluorescence imaging 115. Alternatively, smaller 5.74 nm cationic carbon dots emitting red light were able to cross the intact rat BBB, allowing early diagnosis contrary to contrast agents. The dots accumulated in orthotopic glioma with high tumor to background ratio allowing accurate tumor delineation 116. cRGD functionalized QDs allowed intravital fluorescent imaging of the tumor for prolonged periods of time in an intracranial GBM mouse model, with down to single cell level fluorescence imaging *ex vivo* 117. These dots could potentially allow real-time imaging and visualization of residual tumor for the surgeon or aid in cell identification and localization in biopsies. NIR and (infrared) IR probes have deeper tissue penetration than fluorescent imaging but might still be insufficient for deep seated tumors. One way to increase the imaging possibilities of fluorescent probes beyond the optical diffusion limits is photoacoustic (PA) imaging. This technique takes advantage of the sound waves generated by particles absorbing light, which can be converted into high resolution structural images. Ge *et al.* designed a carbon-based QD that could emit red light. Intravenously injected dots accumulated in xenograft HeLa tumors *via* EPR and allowed *in vivo* fluorescent as well as PA imaging. Moreover, the dots could be used as photothermal inducers, as a large percentage of the absorbed energy is converted to heat, facilitating thermal ablation of tumor tissue 118. The FDA approved fluorescent dye indocyanine green (ICG) has been used in a similar fashion in a theranostic particle for deep seated GBM. Molybdenum disulfide NPs where used to passively target ICG to intracranial glioma in an orthotopic mouse model. Using PA imaging it was possible to identify tumor mass up to 3.5 mm below the scalp 119.

**Figure 4:**
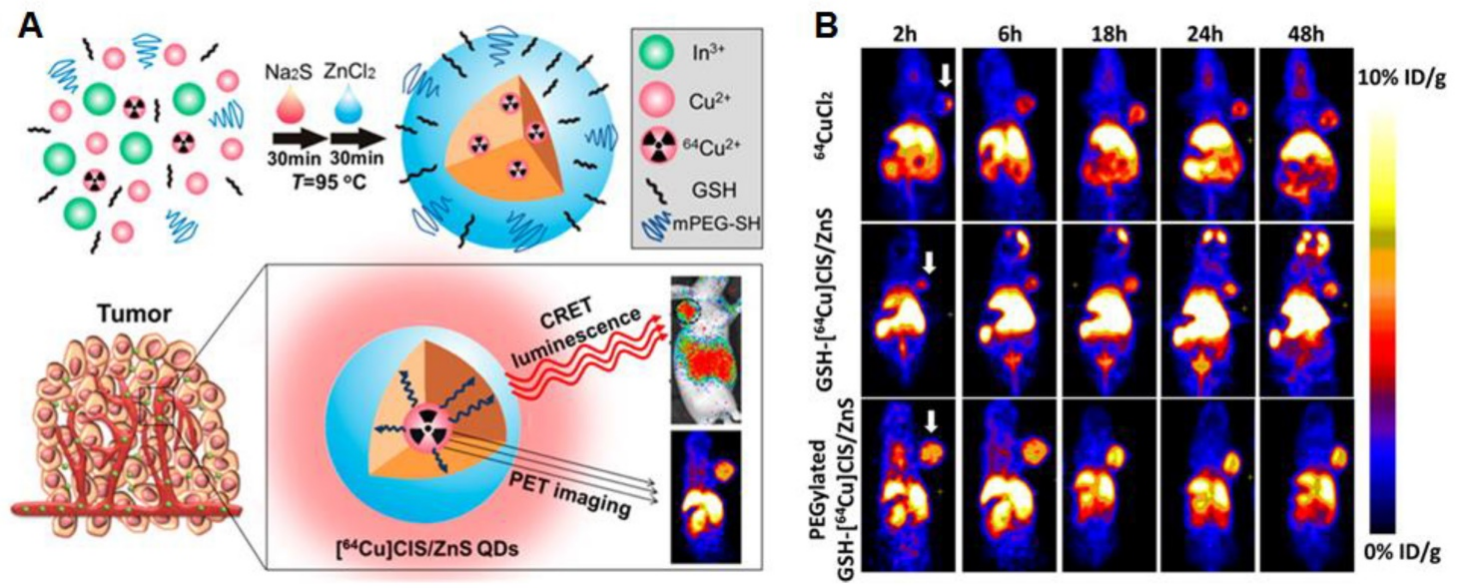
(A) and (B): Radioactive [^64^Cu]CLS/ZnS QDs as PET/self-illuminating luminescence imaging agents show promising *in vivo* visualization possibilities. Adapted with permission from 120, copyright 2014 American chemical society.

Alternatively, QDs can be coupled to other imaging agents such as radioactive tracers to allow deeper tissue imaging. PEGylated radioactive QDs were made using metal chlorides and ^64^CuCl_2_. These particles could be imaged using PET scans in a xenograft GBM mouse model. The dots were self-luminescent *via* cerenkov resonance energy transfer, making it possible to visualize tumors *via* luminescence imaging as the EPR effect resulted in tumor accumulation of the PEGylated particles 120 (Figure 4A and B). Radiolabeled carbon based dots (C-dots) with cRGD targeting showed promising tumor localization and imaging possibilities together with favorable pharmacokinetics and dynamics in human melanoma patients with metastases. The C-dots could be visualized accurately *via* PET imaging and showed promising fluorescence imaging opportunities in an earlier mouse study 121.

**Figure 5:**
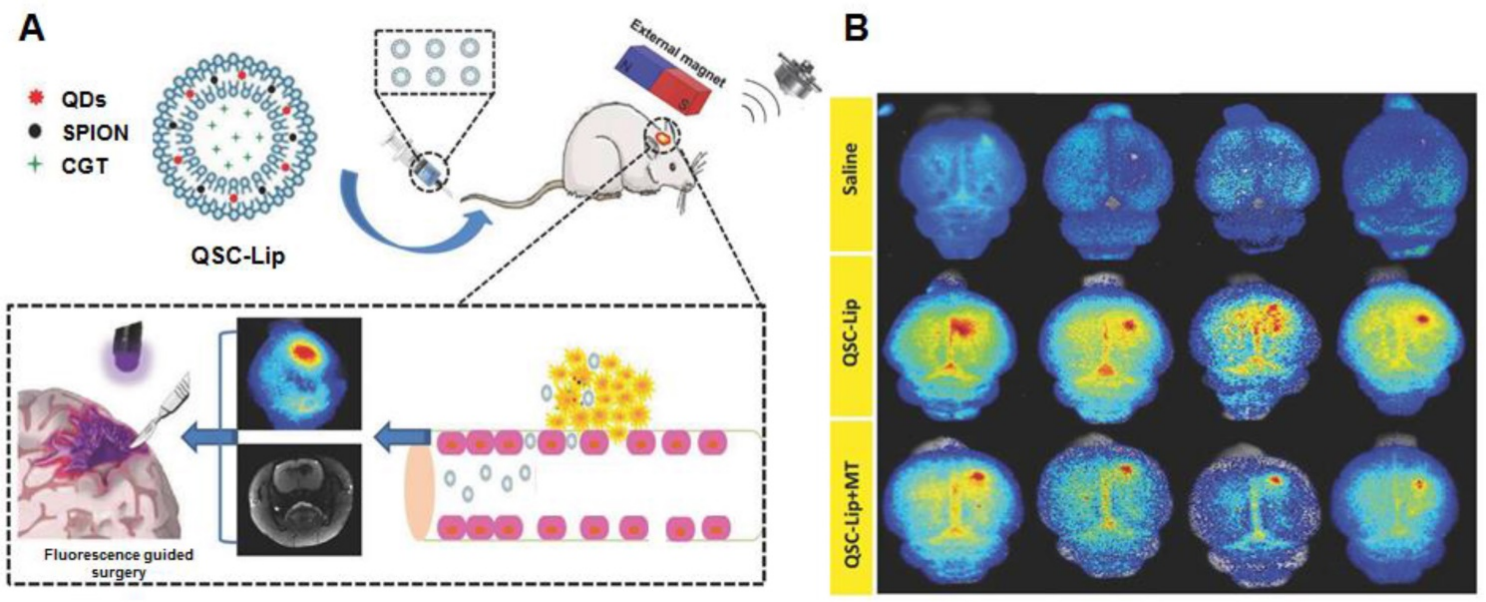
(A) and (B) Using an exogenous magnetic field to target liposomes loaded with multiple imaging agents and therapeutic drugs to an intracranial tumor. The integrated QDs can be used for fluorescence guided resection. Adapted with permission from 123, copyright 2018 WILEY‐VCH Verlag GmbH & Co. KGaA, Weinheim.

As mentioned in the section of active targeting, superparamagnetic iron oxide nanoparticles (SPIONs) are extensively investigated as standalone theranostic particles as well. The magnetic iron core can be visualized *via* MRI allowing tracking of the particles and simultaneously present a way of directing the particles toward their goal *via* magnetic fields. A second biocompatible layer reduces toxicity and extends circulation 122. By including QDs in a liposome loaded with SPION, a dual-imaging platform was created that simultaneously functions as a carrier for therapeutics. The liposomes could be directed to the tumor *via* magnetic targeting, assisted by ultrasound-targeted microbubble destruction of the BBB. They were visualized by MRI (SPION) and fluorescent imaging (using the QD). The fluorescent signal was able to improve gross resection while the loaded cinglitide could inhibit tumor growth 123 (Figure 5A and B). Locally, SPIONs can be used to induce hyperthermia. Aminosilane-coated SPIONs are approved in Europe under the name NanoTherm^®^ for hyperthermal treatment of primary and recurrent GBM. The particles are injected intratumorally, followed by applying alternating magnetic fields to produce cell killing heat. Combined with radiotherapy, NanoTherm^®^ prolonged survival in 66 patients with recurrent GBM 124. Similarly, by coating the cavity wall with magnetic NPs after tumor resection, hyperthermal treatment of residual tumor was possible. Combined with radiotherapy this approach showed a prolonged anti-tumor immune response with some patients achieving long lasting stable disease 125. A hybrid NP, consisting of a magnetic iron core coated with a carbon shell with photoluminescent properties, was used to image and treat mice bearing C6 GBM tumors. The particles could be visualized with fluorescent imaging as well as MRI and by using a NIR, photothermal treatment significantly inhibited tumor growth in tumor-bearing mice 126.

As more and more information is gathered on the presence of different cellular compositions in different tumor regions, subtype specific diagnostic/ theranostic NPs might be used to elucidate the tumor make up and provide tailored therapy 46.

### Considerations for nanocarrier-based drug combination therapy for GBM

Due to differences in BBB permeability, drug stability and pharmacokinetics, therapeutic efficacy may be disappointing when simply administering two or more theoretically synergistic compounds (*e.g.* Bortezomib and HDAC; O^6^-BG and TMZ) 127, 128. Careful planning is needed to make sure both compounds arrive at the target site with desired concentration. Alternatively, encapsulating multiple agents in a single nanocarrier ensures both agents will travel and reach their destination together. A folate-targeted poly (ε-caprolactone) (PCL) and linear poly (ethylene imine) (PEI) cationic delivery system was capable of co-encapsulating BCL-2 siRNA and DOX. Blocking the production of BCL-2 effectively inhibited the anti-apoptotic response and sensitized C6 cells to DOX treatment, demonstrating the synergistic effect of the DOX and BCL-siRNA when they are delivered simultaneously 129. By tweaking the release kinetics of the individual compounds, it is possible to control drug ratios and achieve improved therapeutic effects. By optimizing PTX: TMZ ratios within a single NP, therapeutic efficacy could be improved compared to single drug NPs or free drug combinations against GBM cell lines *in vitro*. Intravenous administration of NPs encapsulating the compounds in their optimal ratio significantly inhibited tumor growth in a U87 subcutaneous model *in vivo* 130.

To find optimized ratios, different approaches to define the combination effect need to be conducted before the nanoformulations. The most well-known median-effect-based method described by Chou and Talalay could be helpful to analyze the drug combination effect, which provides strict quantitative standards to establish drug combination therapy by research institutions, drug inspection bureaus and pharmaceutical factories 131. As for the nanocarrier design, drug coloaded in same nanocarrier might be superior at cellular levels as synergistic drugs can be both released in the same cell. However, a few factors such as the partition coefficient, the molecular weight and the synergistic ratio of the payloads have to be considered. Thus, free drug form or drug encapsulated in different NPs seems to be more flexible for combination regimen design.

Besides the optimal ratios, the timing and order in which drugs are administered can have significant effects on the treatment outcome. For instance, the complex tumor microenvironment can greatly impair drug distribution within the tumor 132. Improved drug distribution and efficacy can be achieved by administering the drugs in a specific order, thereby “priming” the microenvironment. Patients who were treated with the antiangiogenic drug cediranib together with radio-chemotherapy achieved improved PFS and OS when they showed improved tumor perfusion because of the vasculature normalization induced by the anti-angiogenic kinase inhibitor given first 133. Whether the improved survival is due to increased efficacy of the secondary treatment, the additional effect of the anti-angiogenic therapy or both remains unclear 134. However, several clinical studies observed that bevacuzimab only provided survival benefits when combined with radio-chemotherapy 53. This suggests that the anti-angiogenic drug alone was not primarily responsible for the therapeutic effect and was more important in a supportive role to improve drug distribution with possible additional effects on interstitial fluid pressure and hypoxia, two factors that influence both drug distribution and patient survival 53. Another approach addresses the movement of (nano-) particles in the dense tumor interstitial space. Inducing cellular apoptosis can open up this interstitial space, thereby improving tumor penetration and transfection by siRNA NPs 135. Similar results were achieved by attacking structural ECM molecules. Pretreating mice with losartan, a drug against hypertension that additionally acts as an anti-fibrotic agent, reduced collagen I levels and improved the distribution and efficacy of an oncolytic virus and Doxil^®^ (both ~100 nm) in several tumor models 73.

The timing and order of treatments can also significantly affect the way tumors respond to certain insults on a cellular level, a factor that often seems overlooked. Knocking down epidermal growth factor receptor (EGFR) signaling in triple-negative breast cancer cells markedly increased their sensitivity to subsequent DOX treatment. Reverse order or simultaneous administration had no such effect and could even be inhibitory, emphasizing the importance of timing rather than just co-administration 136. Inhibiting the Wnt/β‐catenin signaling pathway with aspirin, sensitized glioma cells to TMZ. Co-loading aspirin with TMZ in a PLGA microsphere significantly increased tumor inhibition compared particles loaded with TMZ alone in a xenograft mouse model 137. Similarly, a wildtype *p53* gene plasmid was loaded in a transferrin targeted liposome (SGT-53) which could sensitize human glioma cells to TMZ. Moreover, pretreatment of TMZ resistant tumors with SGT-53 reversed TMZ resistance, while co-delivery of SGT-53 and TMZ postponed the development of TMZ resistance in an intracranial mouse GBM model 138. Alternatively, a stapled peptide was designed against the *p53* inhibitors MDMX and MDM2 which was loaded in an RGD-targeted micelle. The micelles could reach an intracranial tumor and exert potent *p53*-dependent anti-proliferative activity. In addition, activating *p53* sensitized the tumors for subsequent TMZ treatment 139.

Intelligent designs of NPs 140, 141 or more elaborate DDSs 142 can facilitate such sequential drug exposures and might improve the therapeutic efficacy of compound combinations. For instance, a 1,2-dioleoyl-sn-glycero-3-phosphate (DOPA) and poly L-lactide (PLA)-PEG NPs system loaded with erlotinib and DOX was found to facilitate an optimal order of administration for these two drugs, which is according to the fact that basal A type of triple negative breast cancer cells can be sensitized to DNA-damaging agents after EGFR signaling is suppressed 141. Thus, it could be possible for nanocarriers to not only codeliver different therapeutic compounds with different physicochemical properties, but also sequentially release them in a desired order.

## Perspectives

As single drug therapies often seem insufficient for the treatment GBM, the focus has shifted to combination therapies. It is important to find combinations that act synergistically and preferably on different targets as tumor heterogeneity and emergence of resistance pathways need to be considered. Genetic profiling of tumors can help in finding the most promising approach. Incorporating established therapies in combination approaches will ensure faster translation to the clinic while adding compounds for novel targets such as the tumor immune microenvironment has shown promising results. However, simply administering two compounds systemically will most likely result in underwhelming results. Brain delivery of most compounds has proven challenging and seems to be insufficiently acknowledged as few clinical trials investigate or report brain concentrations of the used compounds.

Intracranial delivery of therapeutics is promising but still needs significant optimization before it is sufficiently practical to be implemented in standard care. Systemic delivery is predominantly hampered by the BBB. There is increasing interest in nanoformulations for the delivery of drugs to the brain and has shown promising results in preclinical (small) animal studies. The ideal design of a nanosized delivery system depends predominantly on the encapsulated molecules, the preferred release profile of each compound (simultaneous or time staggered) and which cells are targeted. Nevertheless, certain characteristics seem to be universally beneficial. PEGylating the particle and keeping the size around 20-75nm ensures prolonged circulation and at the same time allows sufficient tissue penetration. Factors such as the partition coefficient, the molecular weight and the synergistic ratio of the payloads have to be considered as well. Although nanocarrier based brain delivery shows promising results in preclinical *in vivo* studies, results fail to translate to the clinic. This is partly due to too simplistic (murine) models that don't represent the complex human tumor heterogeneity.

In order to have a more accurate prediction of clinical outcome of novel therapeutic strategies, an ideal mouse glioma model should be orthotopic and highly reproducible with predictable tumor growth, bear a genetic similarity to human glioma, show cellular heterogeneity and angiogenic-like growth 143. Patient derived GBM cells can mimic the invasiveness and infiltration behavior of human GBM. Nevertheless, the inability to fully restate the genetic heterogeneity and phenotype of spontaneously occurring human brain tumors in a foreign microenvironment is still a major limitation 144. Mostly, genetically engineered mouse models (GEMMs) have used combinations of the tumor suppressor *p53* and/or *Rb* knock-down and the activation of pro-survival RTK and Ras signaling to allow for *de novo* tumor formation. With GEMMs, it could be possible to investigate the function of tight junction proteins, transporters, or ECM components in BBB development and biology as well. On the other hand, GEMMs also failed to recapitulate the intratumoral heterogeneity seen in patients. Other limitations like expensive breeding and low tumor penetrance have to be considered 145. Canine spontaneous brain tumors are valuable model systems to evaluate the clinical translation of different brain tumor therapeutic strategies for many reasons. The prevalence of malignant gliomas among dogs is comparable to that in humans. The gross pathological, microscopic and immune-histochemical features are similar between human and canine spontaneous brain tumors. The immunologic features associated with human and canine brain tumors also share similarities, such as the tumor infiltration of macrophages or T cells and PD-1 immunoinhibitory mechanisms. Those features are less frequently observed or sometimes absent in rodent human brain tumor xenograft models. Therefore, those models could be considered as a clinical relevant tumor model for investigations of novel drug delivery systems 146.

Additionally, EPR based passive brain targeting of NPs as seen in animal models fails to translate to the clinic. Active targeting is often suggested to improve tumor accumulation yet it is most likely more favorable to pre-select for patients that are suited for NP based treatment. Novel imaging modalities as well as the rise of nanotheranostics will provide the opportunity to pre-select patients with appropriate tumor characteristics such as high tumor perfusion and large relative blood volumes. Additionally, nanotheranostics will provide a way to monitor drug delivery and possibly visualize treatment outcome.

The preclinical success of immunotherapy of GBM has not been replicated in human clinical trials. Both vaccines as single therapy and immune check point inhibitors have led to disappointing clinical results 147, 148. Future immune-based strategies will be focused on combination of different immunotherapeutic approaches to reduce immunosuppression and/or enhance immune response with other modalities.

## Conclusions

GBM is a complicated cancer that involves various sophisticated molecular pathways, gene mutations, and tumor microenvironments. Despite plentiful investigations, an unmet medical need persists for the treatment of invasive GBM. Here we have described the current strategies for nanomedicine-based drug combination therapies. With increasing knowledge of GBM molecular pathways, increasingly more intelligent drug combination strategies will be developed. Nanocarriers will be designed to improve delivery and allow targeting of different pathways within the tumor microenvironment. Future approaches will exploit different nanocarrier-based combinations, the most promising being immunotherapy and nanotheranostics, to enhance the therapeutic benefits for GBM. With the broad knowledge and efforts of researchers, nanocarrier-based combination therapies are expanding the way for success in the battle for GBM treatment.

## Figures and Tables

**Table 1:** Rationale of nanocarrier-based combination therapy against GBM

Issues with single drug treatment	Advantages of combination therapy	Advantages of nanocarrier-based drug delivery	Advantages of nanocarrier-based combination therapy
• Tumor heterogeneity • DNA damage repair • Glioma stem cells • Efflux pump • Dose-limiting toxicity	• Combination of drugs with different mechanisms of action • Combination with anti-GSC drug • Combination with efflux-pump inhibitor • Combination with MGMT inhibitor • Combination of drugs with non-overlapping toxicity	• Drug encapsulation and solubilization • Drug protection • Increase of cellular uptake of nanoparticles *via* endocytosis • Targeted delivery • Controlled/sustained release kinetics • Improvement of drug half-lives • Facilitate diagnostic or theranostic agents	• Combination of drug with different properties (solubility, BBB permeability, pharmacokinetics) • Ensure the synergistic drug ratio • Ensure the colocalization of drugs into tumor site • Facilitate sequential drug exposures

**Table 2:** Recent clinical trials of drug combination for GBM treatment

Drugs	Mechanism of action	Condition	Phase/Status	Major findings	Clinical trial ID
Bevacizumab; Irinotecan	Anti-VEGF antibody; Topoisomerase I inhibitor	Recurrent Gliomas	phase II/Completed in 2013	No results found	NCT00921167
O^6^-Benzylguanine; Temozolomide	O^6^-alkylguanine-DNA alkyltransferase inhibitor; Alkylating agent	Temozolomide- resistant malignant glioma	phase II/Completed in 2008	No results found	NCT00613093
Imatinib; Hydroxyurea	Tyrosine kinase inhibitor; ribonucleoside diphosphate reductase inhibitor	Recurrent/ progressive grade II low-grade Glioma	phase II/Completed in 2012	Groups: patients with astrocytoma or oligodendroglioma; 12-month PFS:44% and 34% respectively	NCT00615927
Cediranib; Lomustine	Tyrosine kinase; Alkylating agent	Recurrent GBM	Phase III/Completed in 2016	Groups: patients received cediranib alone, lomustine alone or drug combination; PFS: 92, 125 and 82 days respectively	NCT00777153
Erlotinib; Vorinostat; Temozolomide	Tyrosine kinase inhibitor; Histone deacetylase inhibitor; Alkylating agent	Recurrent GBM	Phase II/Terminated in 2014 (Unanticipated Toxicities)	No results found	NCT01110876
Sorafenib; Temsirolimus	Tyrosine kinase inhibitor; mTOR inhibitor	Recurrent GBM	Phase I/II/Completed in 2013	Groups: patients not undergoing surgery or received anti-VEGF therapy; 6-month PFS: 17% and 10% respectively	NCT00329719
Bevacizumab; Sorafenib	Anti-VEGF antibody; Tyrosine protein kinases	Recurrent GBM	Phase II/Completed in 2014	Groups: patients received sorafenib high dose or low dose; 6-month PFS: 26% and 17% respectively	NCT00621686
Bevacizumab; Temsirolimus	Anti-VEGF antibody; mTOR inhibitor	Recurrent GBM	Phase II/Completed in 2010	No results found	NCT00800917
Erlotinib; Sirolimus	Tyrosine kinase inhibitor; mTOR inhibitor	Recurrent GBM	Phase II/Completed in 2009	Group: patients received erlotinib and sirolimus; 6-month PFS: 3%	NCT00672243
Vorinostat; Bortezomib	Deacetylase inhibitor; Proteasome inhibitor	Recurrent GBM	Phase II/ Completed in 2010	Groups: patients not undergoing surgery or undergoing surgery; 6-month PFS: 0 and 29% respectively	NCT00641706
Bevacizumab; Erlotinib	Anti-VEGF antibody; Tyrosine kinase inhibitor;	Recurrent GBM	Phase II/ Completed in 2010	Groups: patients with grade III or grade IV malignant glioma; 6-month PFS:44% and 29% respectively	NCT00671970
Temozolomide; SGT-53	Alkylating agent; Liposome-p53 DNA	Recurrent GBM	Phase II/ Recruiting	No results found	NCT02340156
Glasdegib; Temozolomide	Inhibits SHH pathway interfering with cancer stem cells and endothelial migration; Alkylating agent	Newly diagnosed GBM	Phase IB/II/ Recruiting	No results found	NCT03466450
Bortezomib; Temozolomide	Deplete the MGMT enzyme; Alkylating agent	Recurrent GBM with unmethylated MGMT promoter	Phase IB/II/ Recruiting	No results found	NCT03643549
Bevacizumab; Capecitabine	Anti-VEGF antibody; Target myeloid-derived suppressor cells	Recurrent GBM	Phase I/ Recruiting	No results found	NCT02669173
